# Author Correction: Somatic chromosomal engineering identifies BCAN-NTRK1 as a potent glioma driver and therapeutic target

**DOI:** 10.1038/ncomms16187

**Published:** 2018-03-13

**Authors:** Peter J. Cook, Rozario Thomas, Ram Kannan, Esther Sanchez de Leon, Alexander Drilon, Marc K. Rosenblum, Maurizio Scaltriti, Robert Benezra, Andrea Ventura

Nature Communications 8: Article number: 15987; DOI: 10.1038/ncomms15987 (2017); Published: 07 11 2017; Updated: 03 13 2018

In the original version of this Article, financial support was not fully acknowledged. The PDF and HTML versions of the Article have now been corrected to include the following:

‘This work was supported by grant I10-0095 from the STARR foundation.’

