# Supplementary material for: Somatic chromosomal engineering identifies BCAN-NTRK1 as a potent glioma driver and therapeutic target
Source: Nat Commun. 2017 Jul 11;8:15987. doi: 10.1038/ncomms15987 (PMC5508201; doi:10.1038/ncomms15987)
Supplement: Supplementary Information — Supplementary Figures, Supplementary Tables and Supplementary Methods [file ncomms15987-s1.pdf]

Type of file: PDF
Size of file: 0 KB
Title of file for HTML: Supplementary Information
Description: Supplementary Figures, Supplementary Tables and Supplementary Methods

Type of file: pdf
Size of file: 0 KB
Title of file for HTML: Peer Review File
Description:

Supplementary Methods

TUNEL Staining

TUNEL staining was performed using an In Situ Cell Death Detection Kit (Roche) according to the manufacturer's instructions. Briefly, mouse brain cryosections were boiled in 10mM Sodium Citrate supplemented with 0.05% SDS and incubated with TUNEL reaction mixture at 37° C for 60 min protected from light. Sections were washed in PBS, counterstained with 1ug ul^{-1} DAPI for 15 min, and mounted using Fluoroshield (Sigma).

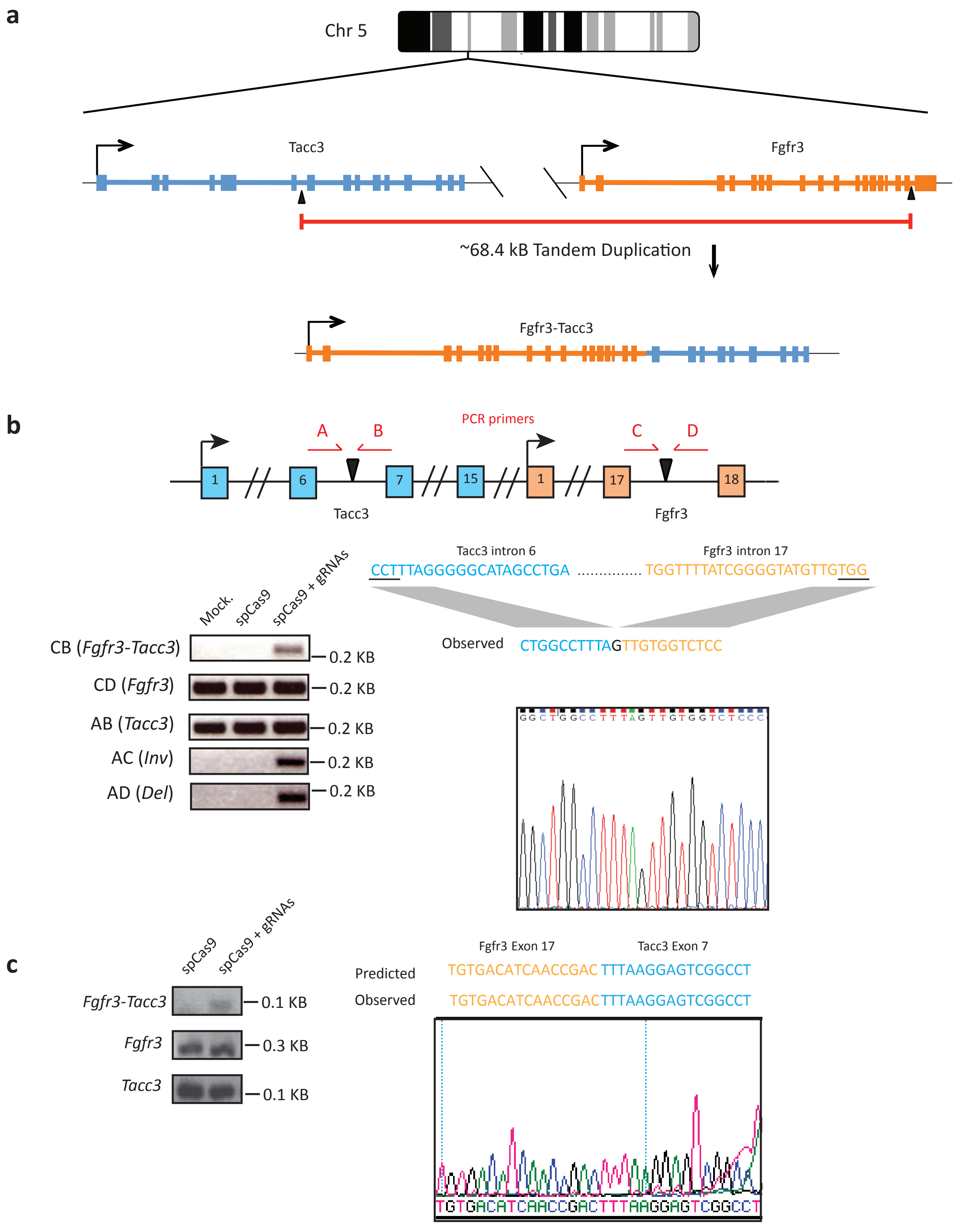

Supplementary Figure 1 - Generation of the Fgfr3-Tacc3 gene fusion

a. Schematics of the region on chromosome 5 encompassing the Fgfr3 and Tacc3 genes and of the Fgfr3-Tacc3 gene fusion resulting from a tandem duplication event. Black arrowheads indicate the genomic breakpoints targeted by the two guideRNAs. **b.** Top, schematic of the targeted intronic regions of Fgfr3 and Tacc3. Arrowheads indicate the gRNA cut sites, while red arrows indicate position and orientation of the primers used to detect the various possible rearrangements. PCRs were performed on genomic DNA extracted from aNSCs nucleofected with the indicated plasmids (bottom left panel). The PCR bands were subcloned and sequenced. A representative chromatogram obtained by sequencing the product of primers CB, detecting the Fgfr3-Tacc3 gene fusion, is shown in the lower right panel. **c**. RT-PCR (left panel) on total RNA extracted from aNSCs nucleofected with Cas9 alone or Cas9+gRNA pair. Primers to detect the wild type Fgfr3 and Tacc3 transcripts or the Fgfr3-Tacc3 fusion transcript were used. The product corresponding to the fusion transcript was subcloned and sequenced. A representative chromatogram is shown in the right panel.

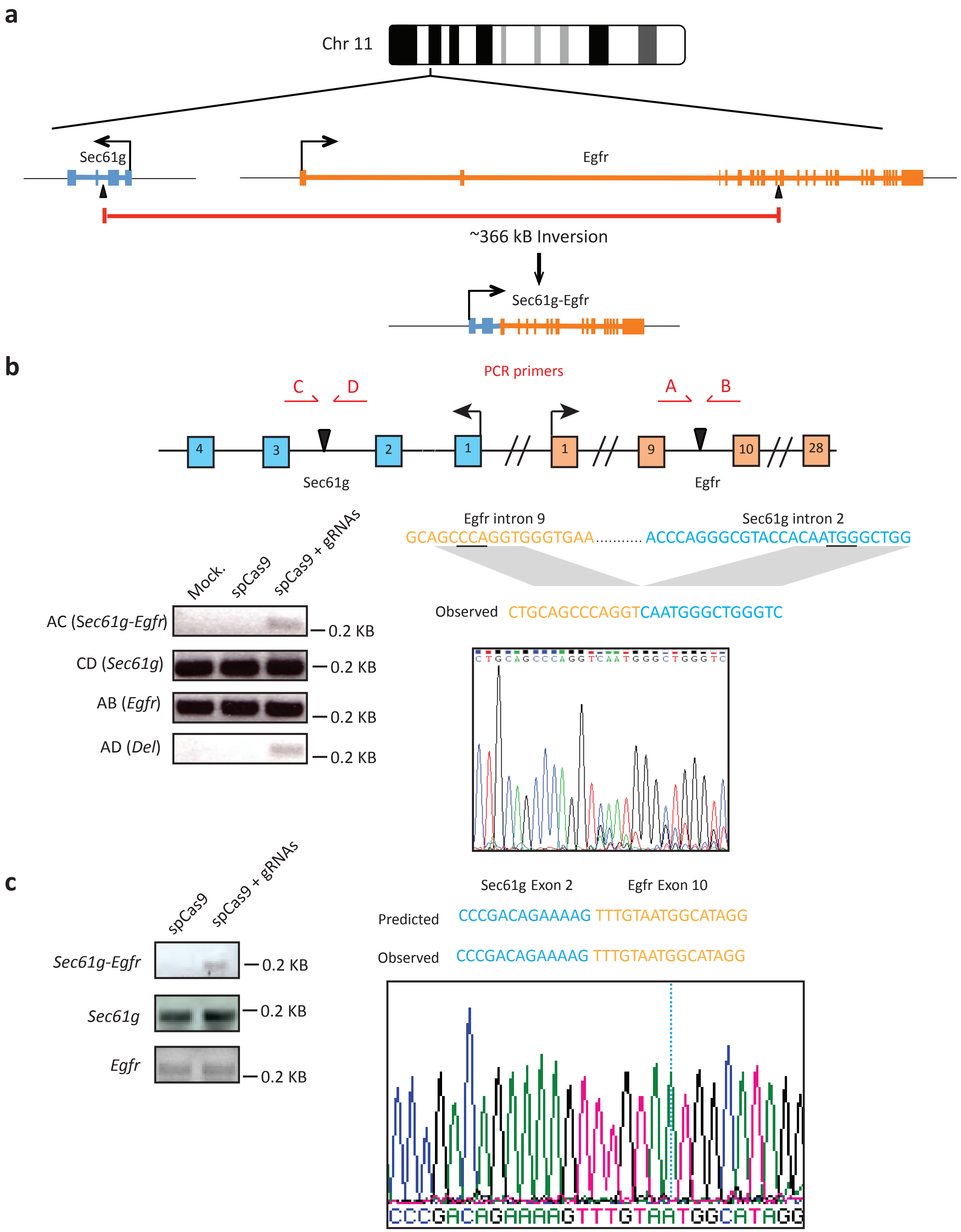

Supplementary Figure 2 - Generation of the Sec61g-Egfr gene fusion

a. Schematics of the region on chromosome 11 encompassing the Egfr and Sec61g genes and of the Sec61g-Egfr gene fusion resulting from a chromosomal inversion. Black arrowheads indicate the genomic breakpoints targeted by the two guideRNAs. **b.**Top, schematic of the targeted intronic regions of Egfr and Secc61g. Arrowheads indicate the gRNA cut sites, while red arrows indicate position and orientation of the primers used to detect the various possible rearrangements. PCRs were performed on genomic DNA extracted from aNSCs nucleofected with the indicated plasmids (bottom left panel). The PCR bands were subcloned and sequenced. A representative chromatogram obtained by sequencing the product of primers AC, detecting the Egfr-Sec61g inversion, is shown in the lower right panel. **c.** RT-PCR (left panel) on total RNA extracted from aNSCs nucleofected with Cas9 alone or Cas9+gRNA pair. Primers to detect the wild type transcripts or the Egfr-Secc61g fusion transcript were used. The product corresponding to the fusion transcript was subcloned and sequenced. A representative chromatogram is shown in the right panel.

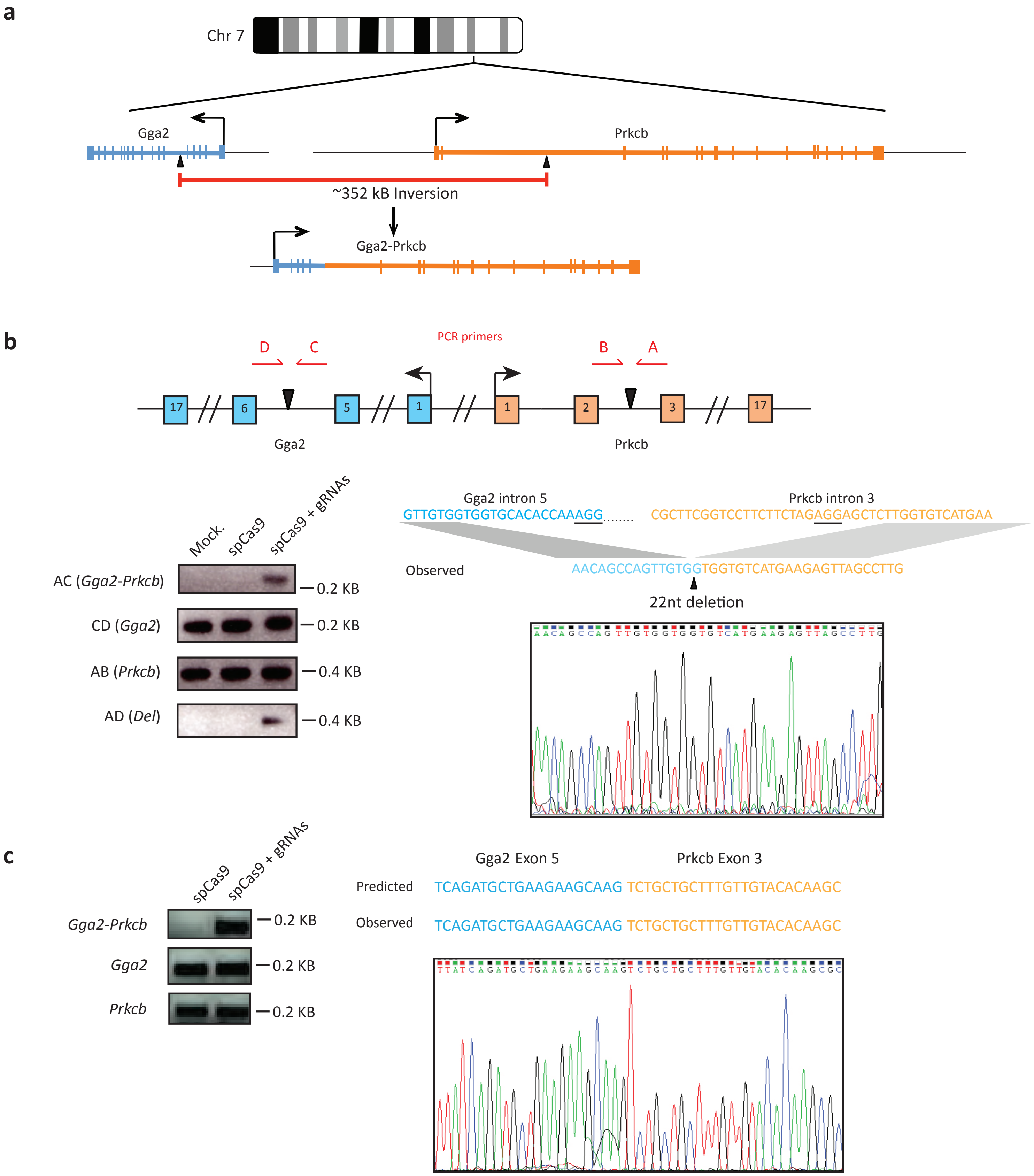

Supplementary Figure 3 - Generation of the Gga2-Prkcb gene fusion

a. Schematics of the region on chromosome 7 encompassing the Gga2 and Prkcb genes and of the Gga2-Prkcb gene fusion resulting from a chromosomal inversion. Black arrowheads indicate the genomic breakpoints targeted by the two guideRNAs. **b.**Top, schematic of the targeted intronic regions of Gga2 and Prkcb. Arrowheads indicate the gRNA cut sites, while red arrows indicate position and orientation of the primers used to detect various possible rearrangements induced by the gRNAs. PCRs were performed on genomic DNA extracted from aNSCs nucleofected with the indicated plasmids (bottom left panel). The PCR bands were subcloned and sequenced. A representative chromatogram obtained by sequencing the product of primers CB, detecting the Gga2-Prkcb gene fusion, is shown in the lower right panel. **c.** RT-PCR (left panel) on total RNA extracted from aNSCs nucleofected with Cas9 alone or Cas9+gRNA pair. Primers to detect the wild type Fgfr3 and Tacc3 transcripts or the GGa2-Prkcb fusion transcript were used. The product corresponding to the fusion transcript was subcloned and sequenced. A representative chromatogram is shown in the right panel.

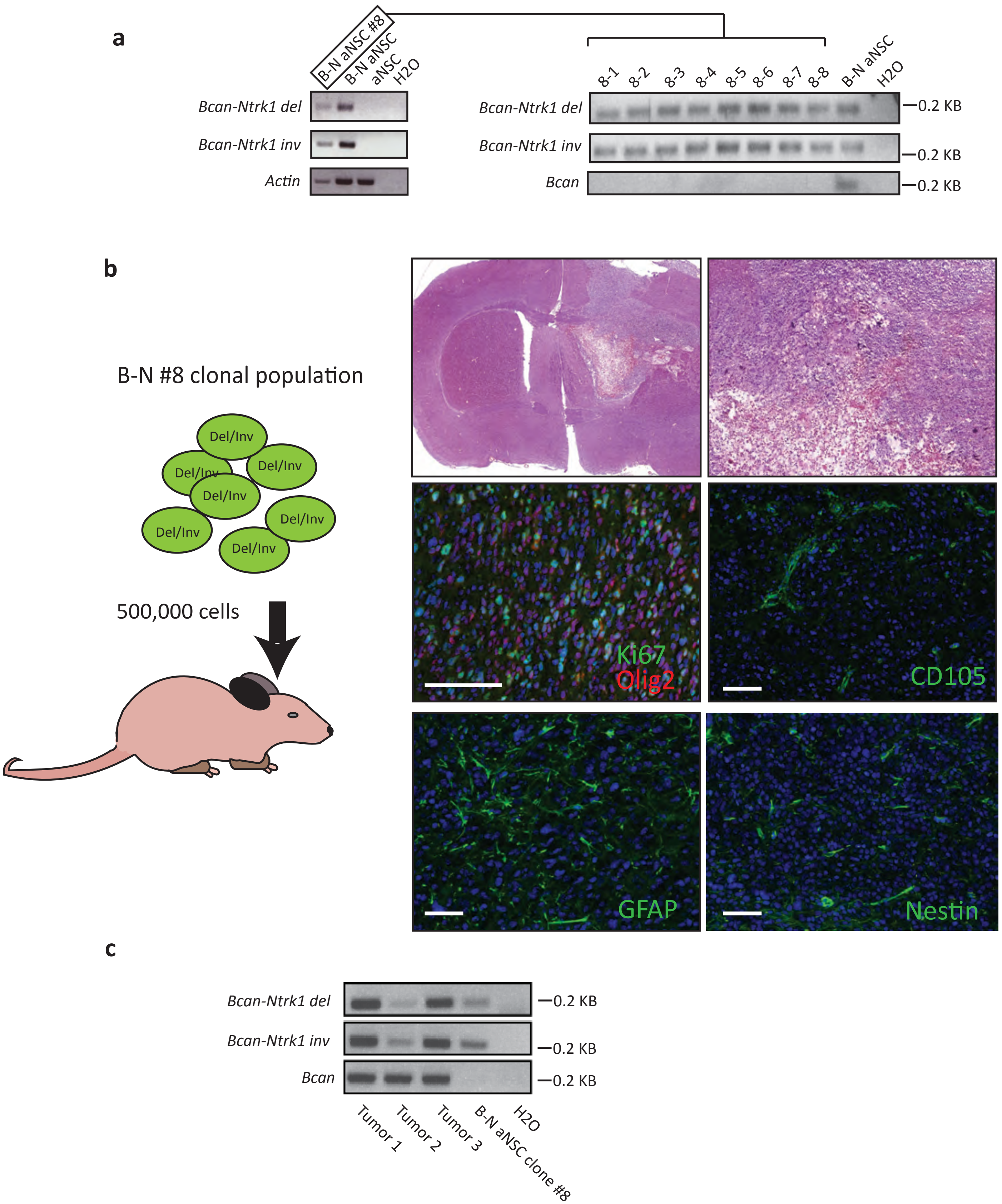

Supplementary Figure 4 - A Bcan-Ntrk1 positive p53-/- aNSC clone produces high grade gliomas in an orthotopic implantation model.

a. PCR genotyping demonstrated p53-/- aNSC clone #8 to be positive for both the Bcan-Ntrk1 deletion and the Bcan-Ntrk1 inversion (right panel). Genotyping of single cell-derived subclones confirmed the clonal nature of this cell line (left panel). **b.** Orthotopic implantation of clonal line #8 into recipient nude mice generated high grade glioma at 100% penetrance that matched the histology and marker expression of tumors derived from the parental and mixed population. Scale bar=0.1mm. **c.** PCR genotyping of bulk tumor tissue revealed the presence of both the Bcan-Ntrk1 deletion and inversion alleles, matching the parental clone. Occurrence of the wild type Bcan allele is predicted to be the result of contaminating non-tumor stromal cells.

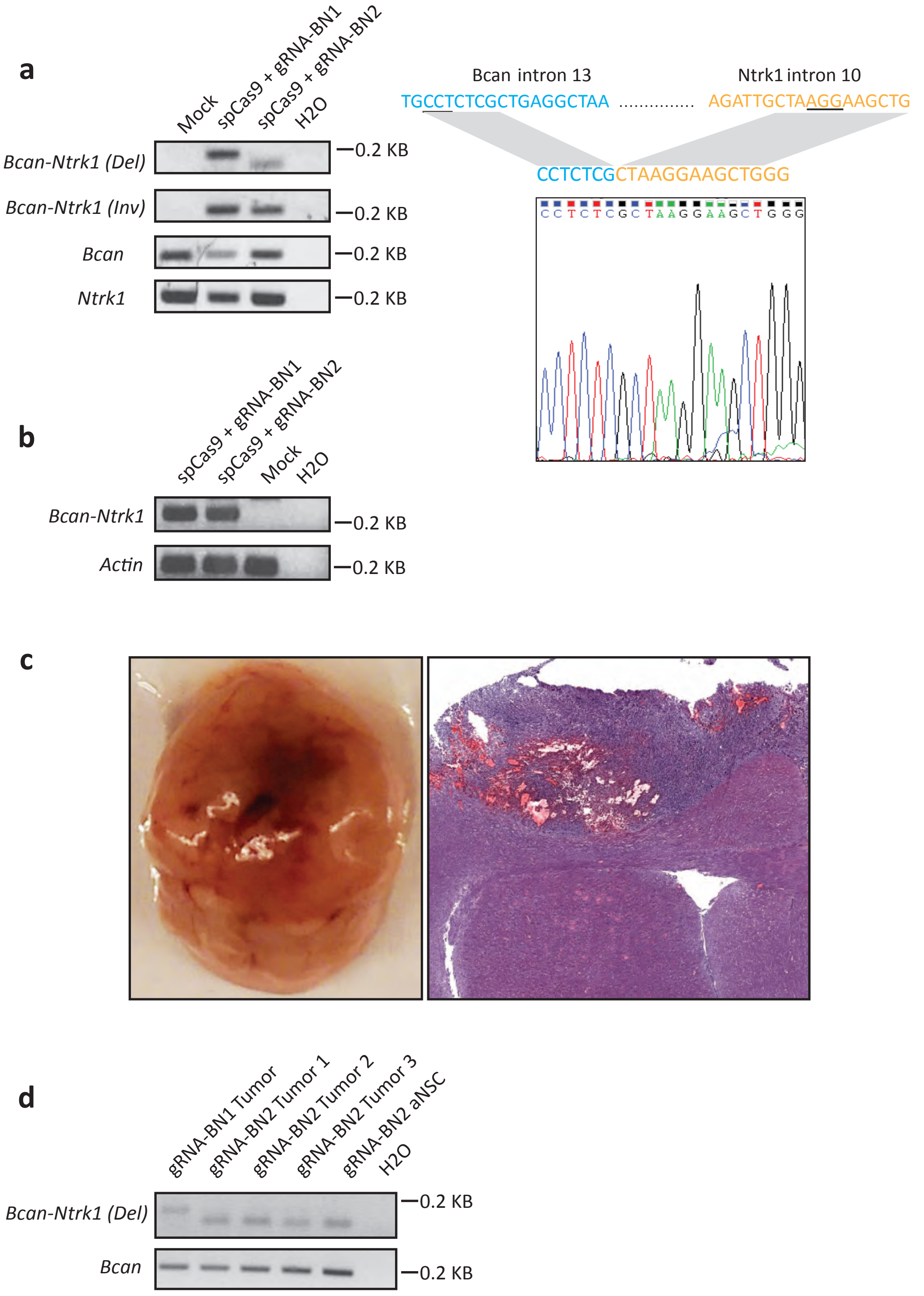

Supplementary Figure 5 - Generation of Bcan-Ntrk1-positive gliomas using a second pair of gRNAs.
a. An alternative gRNA pair (gRNA-BN2) cutting within intron 13 of Bcan and intron 10 of Ntrk1, but with different target sequences than the pair detailed in Figure 1 (gRNA-BN1), were selected and cloned. When delivered to p53-/- aNSC cultures in combination with spCas9, gRNA-BN2 produced a deletion-specific PCR product approximately 50bp smaller than the gRNA-BN1 product, as predicted (left panel). When subcloned and sequenced, this smaller PCR product matched the break-point junction (right panel). **b.** RT-PCR analysis showed an identical Bcan-Ntrk1 mRNA-specific product in cells receiving either BN1 or BN2 gRNAs. **c.** When orthotopically implanted into nude mice, Bcan-Ntrk1 positive p53-/- aNSCs generated using gRNA-BN2 produced brain tumors (left panel) with similar histology to those generated using gRNA-BN1 (right panel). **d.** PCR genotyping of bulk tumor tissue from these tumors showed a Bcan-Ntrk1 deletion-specific band at the predicted size for the BN2 gRNA pair.

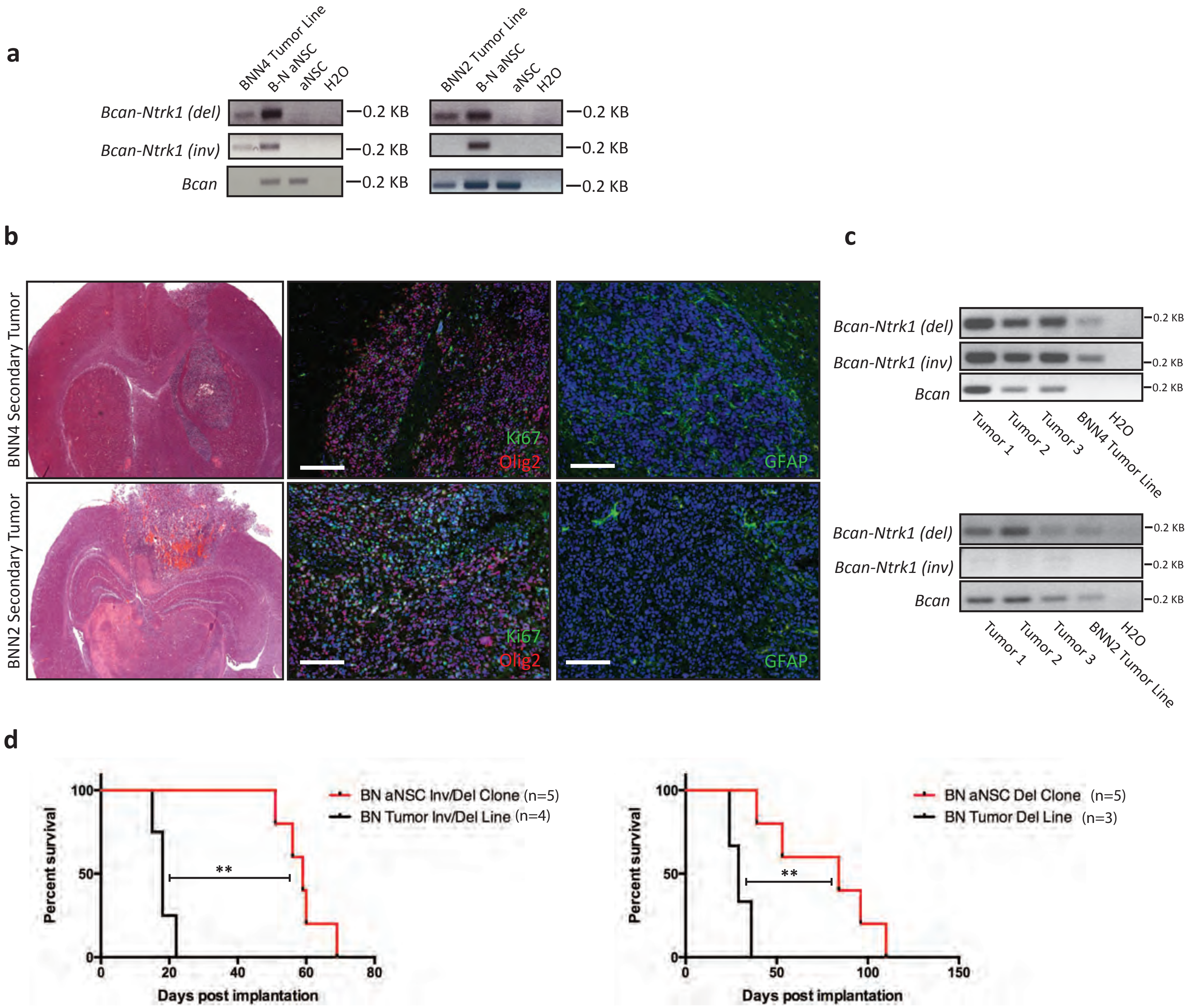

Supplementary Figure 6 – Bcan-Ntrk1 derived tumor cells generate secondary tumors at shortened latency.
a. Primary tumor cultures were established directly from Bcan-Ntrk1 positive mouse tumors generated from implantation of the parental mixed p53-/- aNSC population (Figure 2a). PCR genotyping of 2 of these tumor lines revealed them to be either positive for both the deletion and inversion alleles (BNN4) or solely positive for the deletion allele (BNN2). **b.** Re-implantation of either BNN4 or BNN2 tumor cell lines regenerated high grade glioma with similar pathology to the aNSC-derived tumors. Scale bar=0.2mm. **c.** Genotyping of bulk tumor tissue genomic DNA demonstrated that secondary tumors retained the genotype of the parental tumor cell line. Re-appearance of the wild-type Bcan allele in BNN4 derived tumors is attributed to contaminating gDNA form non-tumor stromal cells. **d.** Primary tumor cell lines generated secondary tumors at greatly reduced latency in comparison to clonal p53-/- aNSCs of matched genotype. **, p<0.01.

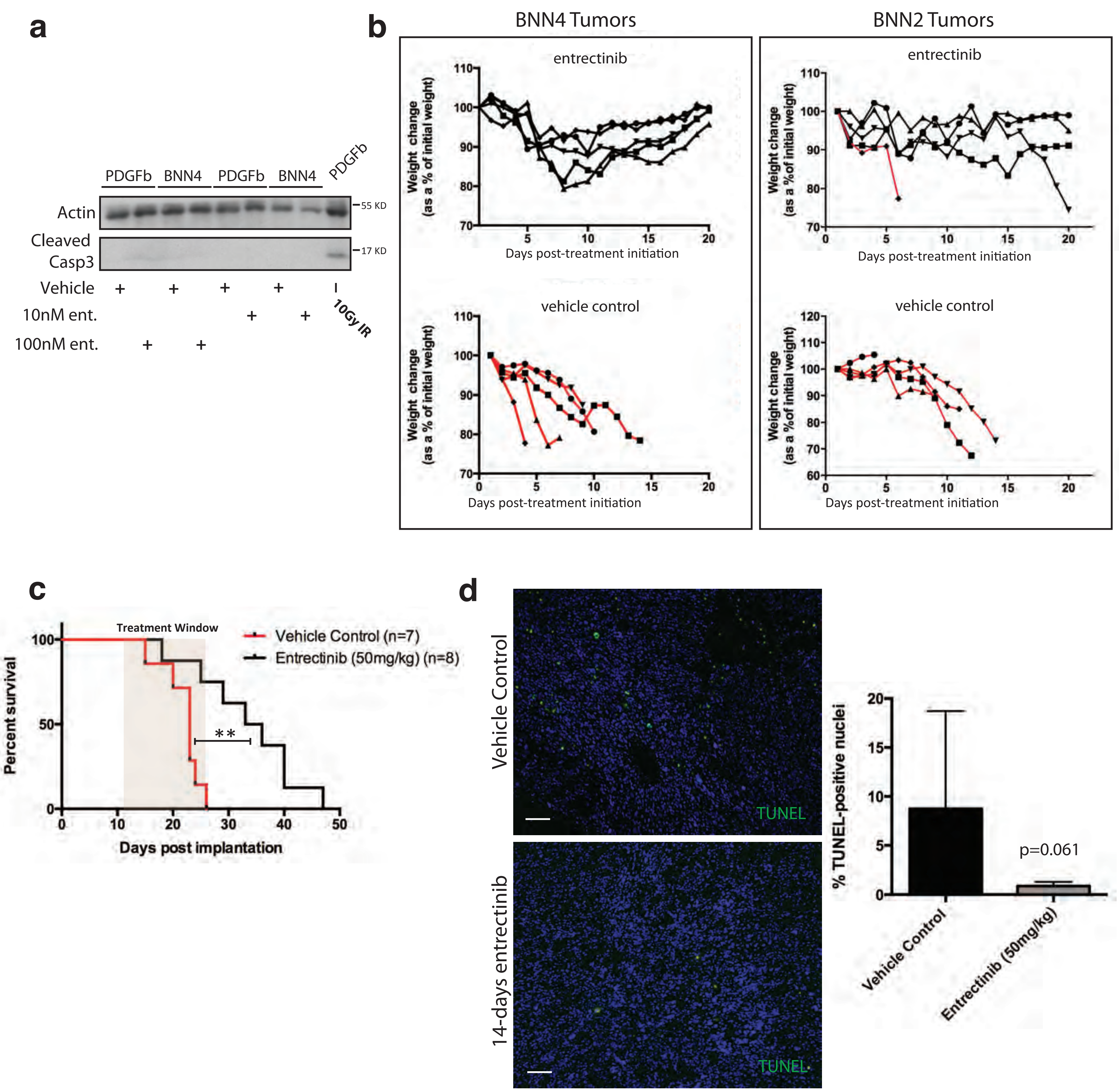

Supplementary Figure 7 – Entrectinib treatment prolongs survival and reduces weight loss of mice harboring Bcan-Ntrk1-driven gliomas without increasing apoptosis of tumor cells.
a. BNN4 or control PDGFb tumor cells were treated with entrectinib or vehicle after 24h growth factor withdrawal and Casp-3 cleavage was assessed after 2h in comparison to PDGFb cells treated with 10 Gy ionizing radiation (IR). **b.** Starting at treatment day 1 (day 12 post-tumor implantation) total body weight was monitored daily for mice treated with entrectinib or vehicle, with the percentage of weight change being calculated each day for each animal relative to the starting weight. Red lines indicate animals sacrificed during the 20 day weight monitoring period. **c.** Mice were injected with BNN2 Bcan-Ntrk1 positive glioma cells on day 0. Daily treatment with entrectinib or vehicle was initiated on day 12 and continued for 14 days. Kaplan-Meier curve of mice receiving entrectinib or vehicle is shown (p-value=log-rank test). **d.** TUNEL staining was performed on BNN4 tumors from mice sacrificed on treatment day 14. Scale bar=0.1mm. Error bars=mean +/- SEM.

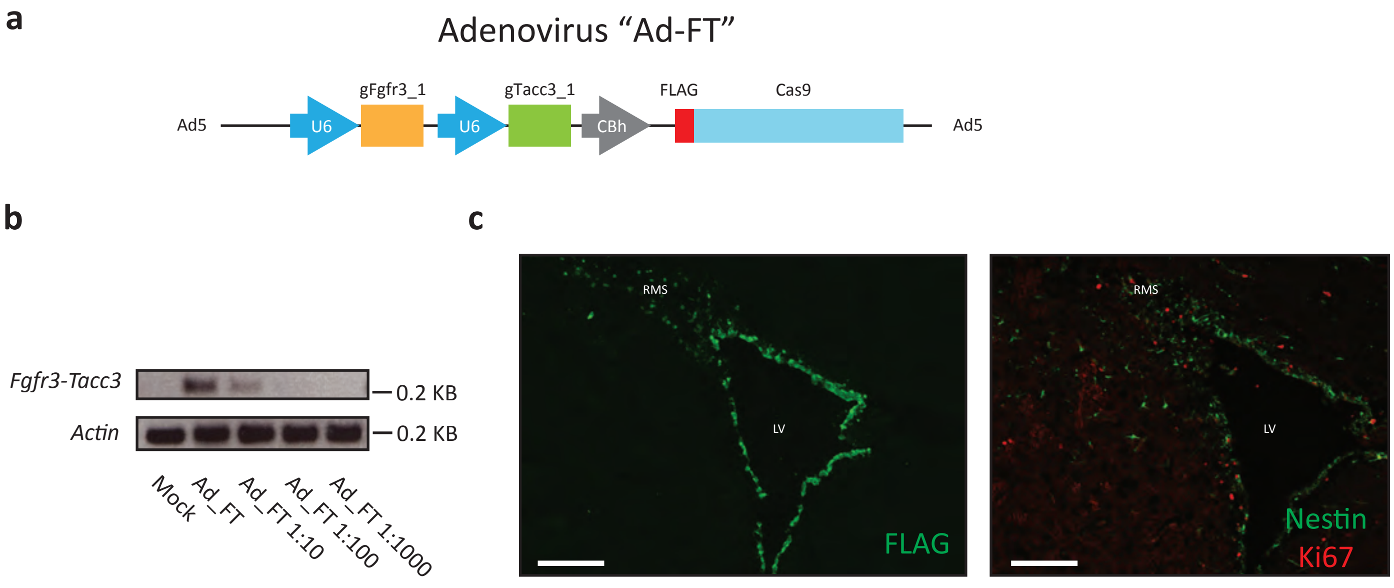

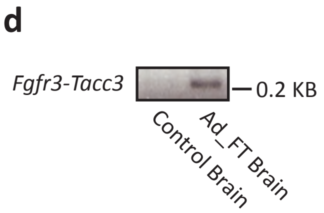

e

Infection	Tumor Bearing animals at day 200
Ad_FT, Ad_CRE	0/5
Ad_CRE	0/4

Supplementary Figure 8 – Direct in vivo engineering of the Fgfr3-Tacc3 rearrangement.
a. Schematic of the recombinant adenoviral vector used to engineer the Fgfr3-Tacc3 rearrangement in vivo. **b.** Mouse NIH-3T3 fibroblasts were infected with a series of dilutions of the Ad-FT virus and subsequently tested for induction of the rearranged products by PCR analysis. **c.** Adult wild type mice were infected with Ad-FT via intracranial injection targeting the neurogenic region of the lateral ventricle. Analysis of infected brains 48 hours post-injection revealed strong expression of FLAG-Cas9 in the region of the lateral ventricle and the rostral migratory stream. Scale bar=0.2mm. **d.** Purified genomic DNA from Ad-FT infected wild type brains was confirmed to be positive for he Fgfr3-Tacc3 fusion allele by PCR. **e.** Summary of in-vivo tumor development from p53f/f animals infected with either a 1:1 mix of Ad-FT/Ad-CRE or Ad-CRE alone.

Supplementary Figure 9. Uncropped images for immunoblots

Fig. 4b

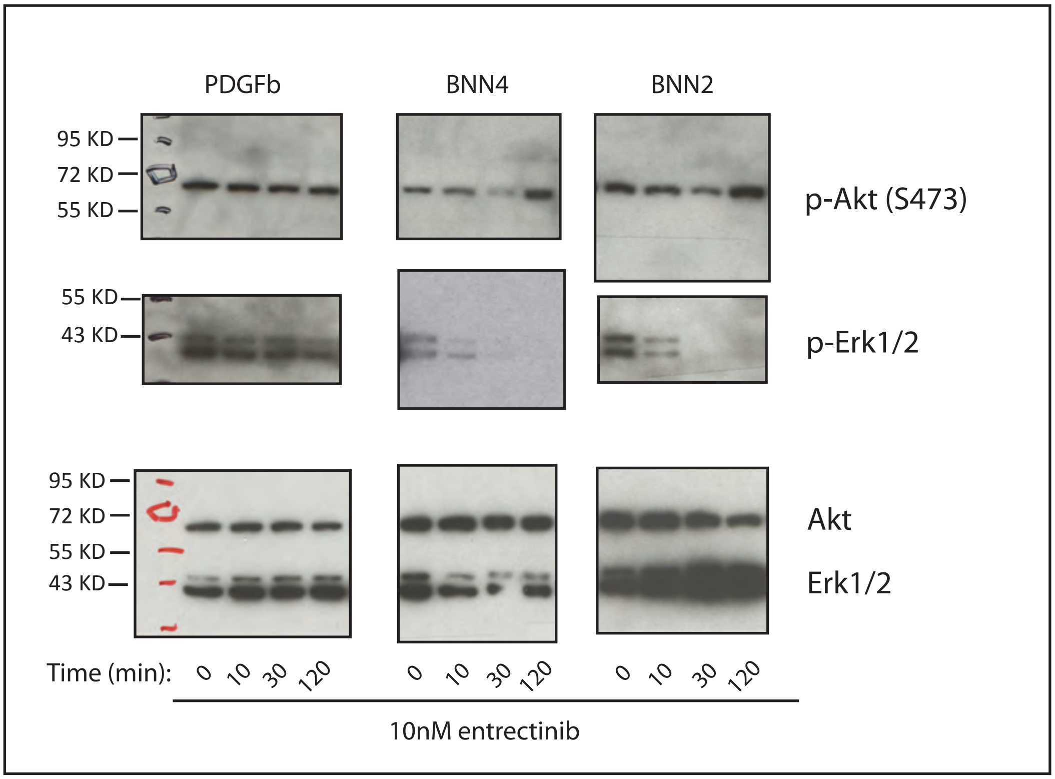

Supplementary Fig. 7a

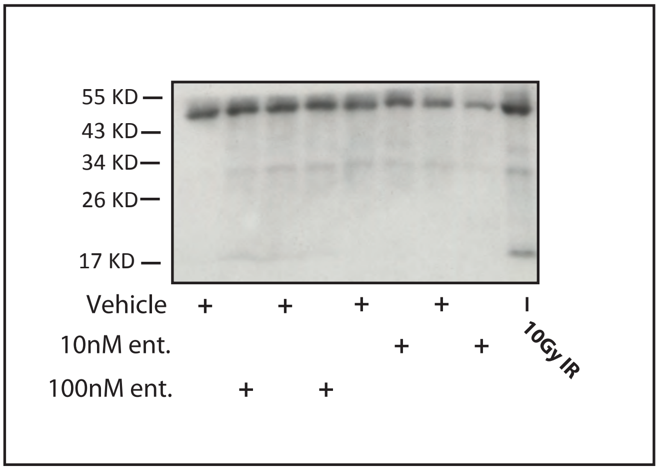

Supplementary Table 1 - Orthotopic implantation summary

Cell Type	Rearrangement	Clone	Number Implanted	Tumor Formation
p53-/- aNSC	Bcan-Ntrk1	non-clonal	500,000	4/5
p53-/- aNSC	Bcan-Ntrk1	non-clonal	200,000	3/3
WT aNSC	Bcan-Ntrk1	non-clonal	200,000	0/4
p53-/- aNSC	Bcan-Ntrk1	#8 (deletion/inversion)	500,000	5/5
p53-/- aNSC	Bcan-Ntrk1	#5 (deletion)	500,000	5/5
p53-/- aNSC	Bcan-Ntrk1	#9 (inversion)	500,000	0/5
p53-/- aNSC	Sec61g-Egfr	non-clonal	500,000	0/5
p53-/- aNSC	Fgfr3-Tacc3	non-clonal	500,000	0/4
p53-/- aNSC	Fgfr3-Tacc3	#8-12 (duplication)	500,000	0/4
p53-/- aNSC	Gga2-Prkcb	non-clonal	500,000	0/4
p53-/- aNSC	Gga2-Prkcb	#8 (inversion)	500,000	0/4
p53-/- aNSC	control (Cas9)	non-clonal	500,000	0/5
WT aNSC	control (Cas9)	non-clonal	200,000	0/4

Supplementary Table 2 - gRNA target sequences used in this study

Name	Gene	Genomic Target Sequence
sgFgfr3_1	Fgfr3	GTTCAGGCTATGCCCCCTAA
sgTacc3_1	Tacc3	GGTTTTATCGGGGTATGTTG
sgBcan_1	Bcan	GTGGAGCTCTGGAGACCTCGT
sgBcan_2	Bcan	GCTTCTCTTAGCCTCAGCGAG
sgNtrk1_1	Ntrk1	GCATGCAGTTGACCAAGCTC
sgNtrk1_2	Ntrk1	GCCGGCTAGCTGAGATTGCTA
sgGga2_1	Gga2	GTTGTGGTGGTGCACACCAA
sgPrkcb_1	Prkcb	CGCTTCGGTCCTTCTTCTAG
sgSec61g_1	Sec61g	GAACCCAGGGCGTACCACAA
sgEgfr_1	Egfr	GCTACAACAGTTCACCCACC

Supplementary Table 3 - Oligonucleotides used in this study

Primer Name	Sequence (5'-3')	Expected WT genomic product
Bcan - A	tatgcctgccattcactgac	206bp
Bcan - B	ggcgctcctccttaataacc	
Ntrk1 - C	agtgtggggtatggaagctg	200bp
Ntrk1 - D	ccattctacaggggactcca	
Tacc3 - A	agcagagttctgacccagga	194bp
Tacc3 - B	caggccgactccttaaactg	
Fgfr3 - C	ggatttagaccgcatcctca	187bp
Fgfr3 - D	actgtccctttgcattcacc	
Egfr - A	ctcttatggccacctccaaa	277bp
Egfr - B	acttgggctgtccttgtcac	
Sec61g - A	aagcaggagctgatgcagag	199bp
Sec61g - B	aaaaacccaactgaaaaatgc	
Prkcb - A	taaaggcagttcccatgtcc	381bp
Prkcb - B	ggcctttgctcatactggtc	
Gga2 - C	gctaaggattgctggtggtc	210bp
Gga2 - D	cctcttgaagggcagccta	
RT-PCR primers		
**Primer Name**	**Sequence (5'-3')**	**Expected fusion cDNA product**
Bcan_RT_For	AGCTACCCCTGGCTCAAATA	226 bp
Ntrk1_RT_Rev	AGGAGGGCAGAAAGGAAGAG	
Gga2_RT_For	CTGGGCTACGGAGAAGGTTA	177bp
Prkcb_RT_Rev	GCAGGAGAACGTGACGAAC	
Sec61g_RT_For	TCAGGTAATGCAGTTTGTGGA	204bp
Egfr_RT_Rev	ACTGGCAGGATGTGAAGGTC	
Fgfr3_RT_For	GGATTTAGACCGCATCCTCA	110bp
Tacc3_RT_Rev	GCAAAGGGCTGTCTTTCAGA	
